# Measurement of Telomere Length for Longitudinal Analysis: Implications of Assay Precision

**DOI:** 10.1093/aje/kwab025

**Published:** 2021-02-10

**Authors:** Daniel Nettle, Shahinaz M Gadalla, Tsung-Po Lai, Ezra Susser, Melissa Bateson, Abraham Aviv

**Keywords:** assay precision, leukocyte telomere length, longitudinal studies, measurement error, quantitative polymerase chain reaction, Southern blot, telomere length, terminal restriction fragment

## Abstract

Researchers increasingly wish to test hypotheses concerning the impact of environmental or disease exposures on telomere length (TL), and they use longitudinal study designs to do so. In population studies, TL is usually measured with a quantitative polymerase chain reaction (qPCR)-based method. This method has been validated by calculating its correlation with a gold standard method such as Southern blotting (SB) in cross-sectional data sets. However, in a cross-section, the range of true variation in TL is large, and measurement error is introduced only once. In a longitudinal study, the target variation of interest is small, and measurement error is introduced at both baseline and follow-up. In this paper, we present results from a small data set (*n* = 20) in which leukocyte TL was measured twice 6.6 years apart by means of both qPCR and SB. The cross-sectional correlations between qPCR and SB were high at both baseline (*r* = 0.90) and follow-up (*r* = 0.85), yet their correlation for TL change was poor (*r* = 0.48). Moreover, the qPCR data but not the SB data showed strong signatures of measurement error. Through simulation, we show that the statistical power gain from performing a longitudinal analysis is much greater for SB than for qPCR. We discuss implications for optimal study design and analysis.

## Abbreviations

ANCOVA: analysis of covariance
IQR: interquartile range
LTL: leukocyte telomere length
ΔLTL: change in leukocyte telomere length
qPCR: quantitative polymerase chain reaction
SB: Southern blotting
TL: telomere length

##  

Recent decades have witnessed an explosion of telomere epidemiology research. Average telomere length (TL), usually measured in leukocytes (hence leukocyte telomere length (LTL)), has been associated with a wide range of environmental exposures, diseases, and psychosocial parameters (see Pepper et al. (1) for a review). The first wave of such studies was almost entirely cross-sectional, but as the field has matured, attention has turned to longitudinal studies. As well as being more informative about possible causal relationships (2, 3), longitudinal studies are potentially more statistically powerful for testing hypotheses in telomere epidemiology (4). The key difference between a cross-sectional study and a longitudinal study, from a purely analytical standpoint, is that in the former, the target parameter is average LTL, whereas in the latter, it is the average change in LTL within individuals over time. There is a large range of variation in LTL between individuals. This variation is stable over time in adulthood and is substantially heritable (5, 6), but it is effectively noise with respect to many of the hypotheses researchers wish to investigate. It is controlled for in longitudinal studies by making within-individual comparisons.

In telomere epidemiology, LTL is typically measured using a quantitative polymerase chain reaction (qPCR)-based relative TL measurement technique (7). This is an inexpensive and high-throughput method allowing for large-scale studies. The method estimates the amount of the telomeric DNA sequence present in a sample (T), relative to the amount of a single-copy gene sequence whose copy number in the genome does not vary (S). Validation of the qPCR method has been demonstrated by correlating T/S values for a set of samples with LTL measured by a “gold standard” method, usually Southern blotting (SB). When measurement is performed in experienced laboratories, these correlations can be high (*r* ≥ 0.85) (7–9). Researchers therefore reason that qPCR LTL measurement captures substantially the same variation as the gold standard. However, demonstrating that qPCR measurements are highly correlated with SB for a cross-section of individuals does not guarantee that the 2 methods will capture the same variation in a longitudinal analysis or that they are equally powerful for testing hypotheses about environmental and other effects on LTL. First, in a longitudinal analysis there are at least 2 LTL measurements, baseline and follow-up. Thus, the measurement error is introduced twice. As long as these errors are uncorrelated, the spurious variance introduced is twice as large as that in a cross-sectional analysis. Second, in a cross-section of individuals, the range of true biological variation is very large: The standard deviation of LTL across individuals in adult humans is about 700 base pairs, with the most extreme individuals differing by 3,000–4,000 base pairs (4, 10, 11). However, much of this variation, reflecting individual differences in TL that are already evident at birth, is irrelevant to hypotheses about environmental or aging effects on telomere dynamics in adulthood. The change in LTL over time within adult individuals is only around 25–30 base pairs/year, on average (10, 11). This means that even an exposure that doubles the rate of telomere attrition will only change average LTL by a few tens of base pairs per year. This is a very small target relative to the range of variation in LTL. Accordingly, the effective precision of qPCR to detect effects of an exposure on telomere dynamics may be much lower than the high cross-sectional correlation with SB seems to imply.

In 2-measurement longitudinal LTL studies where the effect of some exposure or treatment *X* is of interest, researchers have a number of options for data analysis strategy (12, 13). One strategy is to simply test whether LTL at the final time point differs by *X*. This ignores the baseline information, in effect treating the longitudinal study as a cross-sectional one. We henceforth refer to it as the *cross-section approach*. A second strategy (henceforth called the *difference score approach*) is to calculate change in leukocyte telomere length (ΔLTL), the difference in LTL between baseline and follow-up, and test whether ΔLTL differs by *X*. Finally, the *analysis of covariance* (ANCOVA) strategy tests whether LTL at follow-up differs by *X*, with LTL at baseline included in the model as a covariate (though this approach produces biased estimates when baseline LTL is associated with *X* and there is measurement error; see Bateson et al. (14), Oakes and Feldman (15), and the Discussion section).

The relative statistical powers of the 3 approaches depend on the correlation between baseline and follow-up LTL (13). If this correlation is close to 0, the cross-section approach is more powerful than the difference score and as powerful as ANCOVA, whereas if the correlation is high, the power of the cross-section approach is much lower than that of the other two. The correlation between baseline LTL and follow-up LTL is generally lower for qPCR than for SB, exactly because the measurement error is greater (11, 16). Thus, the relative power advantages of the different analysis strategies may be different for SB and qPCR data.

Here, we investigate for the first time the validity of qPCR measurement for capturing longitudinal LTL dynamics and consider the potential implications of the findings for study design and analysis. We present results for a small longitudinal data set of 20 men whose LTL was measured twice, 6.6. years apart, by both techniques. We investigate the correlation between qPCR and SB not just for LTL at baseline and follow-up but also for ΔLTL. Further, we compare the qPCR and SB data for known signatures of measurement error—namely a strong apparent dependence of ΔLTL on baseline LTL due to regression to the mean (16, 17) and a substantial fraction of individuals whose LTL appears to lengthen rather than shorten over time (10). We expect these signatures to be much more marked for the qPCR data than the SB data. We then simulate data sets with the same cross-sectional correlations between qPCR and SB at baseline and follow-up as our empirical data have. This allows us to verify that the observed features of the qPCR estimates of TL dynamics are not quirks of 1 small data set but should be expected more generally. Finally, we use the simulated data sets to investigate the implications of qPCR’s lower precision for statistical power to detect an effect on TL dynamics.

## METHODS

### Empirical data set

We used blood samples from the Stony Brook University biorepository (State University of New York at Stony Brook, Stony Brook, New York). The samples were collected on 2 occasions, baseline and follow-up, 6.6 (standard deviation, 0.5) years apart (2012–2019), for 20 white males aged 53.8 (standard deviation, 4.3) years at follow-up. Donors consented to participate, and institutional review board approval was obtained. LTL was measured at both baseline and follow-up by means of both qPCR and SB independently and blindly in different laboratories, by qPCR at the National Cancer Institute’s Cancer Genomics Research Laboratory (Bethesda, Maryland), and by SB at the laboratory of the Center of Human Development and Aging at Rutgers University (Newark, New Jersey). Each laboratory followed its standard TL measurement protocol (18, 19).

### LTL measurements

DNA was extracted using the Gentra Puregene Blood Kit (Qiagen Inc., Valencia, California), and all samples passed DNA integrity tests (18). For SB, a cocktail of the restriction enzymes *Hin*fI (10 U) and *Rsa*I (10 U) was used to generate the terminal restriction fragments. Measurements were carried out in duplicate and resolved on different gels. The intraclass correlation coefficient for duplicates was 0.93 (95% confidence interval: 0.87, 0.96). Digested DNA samples and DNA ladders were resolved on 0.5% agarose gels. After 16 hours, the DNA was depurinated for 15 minutes in 0.25N hydrochloric acid, denatured for 30 minutes in 0.5M sodium hydroxide/1.5M sodium chloride, and neutralized for 30 minutes in 0.5M tris(hydroxymethyl)aminomethane (Tris), pH 8/1.5M sodium chloride. The DNA was transferred to a positively charged nylon membrane (Roche, Inc., Basel, Switzerland) for 1 hour using a vacuum blotter. Membranes were hybridized at 65^o^C with the digoxigenin-labeled telomeric probe as previously described (18). The digoxigenin-labeled probe was detected by digoxigenin luminescence and exposure on radiographic (x-ray) film.

**Figure 1:**
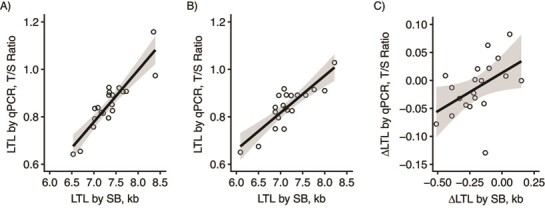
Relationships between leukocyte telomere length (LTL) parameters as measured by quantitative polymerase chain reaction (qPCR) and Southern blotting (SB). A) Cross-sectional correlation between qPCR and SB at baseline (*r* = 0.90, *P* < 0.001); B) cross-sectional correlation between qPCR and SB at follow-up (*r* = 0.85, *P* < 0.001); C) correlation of change in LTL (ΔLTL) from baseline to follow-up by qPCR and by SB (*r* = 0.48, *P* = 0.03). Circles represent individual data points, black lines represent linear fits, and shading shows the 95% confidence intervals for the linear fits. Blood samples were obtained from the Stony Brook University biorepository, Stony Brook, New York, 2012–2019. kb, kilobases; T/S ratio, ratio of telomere signal concentration (T) to that of the single-copy gene (S) (*36B4*).

For qPCR, we used the monoplex method adopted from Callicott and Womack (20). Details have been provided elsewhere (19). Briefly, polymerase chain reaction telomere primers were *Telo_FP* (5′-CGGTTT(GTTTGG)5GTT-3′) and *Telo_RP* (5′-GGCTTG(CCTTAC)5CCT-3′). Primers for the single-copy gene (*36B4*) were *36B4_FP* (5′-CAGCAAGTGGGAAGGTGTAATCC-3′) and *36B4_RP* (5′-CCCATTCTATCATCAACGGGTACAA-3′). The ratio of telomere signal concentration (T) to that of the single-copy gene (S) (*36B4*), or T/S ratio, was normalized using the average T/S ratio obtained from the internal quality control calibrator samples on the same plate. All telomere and *36B4* reactions were run in triplicate, and the average of the measurements was used for all calculations. The intraclass correlation coefficient of the T/S ratios produced by treating each triplicate measurement separately rather than averaging was 0.81 (95% confidence interval: 0.70, 0.89).

### Simulations

We created simulation code to produce data sets that shared key properties with our empirical one. Simulation code runs in R (R Foundation for Statistical Computing, Vienna, Austria) (21) and is available via the Zenodo repository (European Organization for Nuclear Research, Geneva, Switzerland) (22). In each simulated data set, baseline (SB) LTL and ΔLTL were each drawn from distributions with the mean and standard deviation observed in the empirical SB data. We then generated qPCR values at each of baseline and follow-up whose correlations with SB were as observed in the empirical data.

Using the simulation code, we first created 1,000 data sets of the same sample size as the empirical data, to investigate the extent to which the empirical patterns should be expected to recur in other samples. Next, we simulated 1,000 data sets at each of a range of sample sizes (*n* = 20–1,000 people), where a predictor variable *X* with a true effect on telomere attrition was applied to half of the individuals. The scenario we had in mind was an experiment or randomized controlled trial. Thus, we assumed that there was no association between *X* and baseline LTL and that individuals differing in *X* were not different in any other systematic way relevant to their LTL dynamics. We then analyzed the simulated data sets using each of the 3 data analysis strategies described in the Introduction (cross-section, difference score, and ANCOVA), to establish the statistical power of each strategy to detect the effect of *X* at *P* < 0.05. This power analysis was applied to both the simulated SB values and the simulated qPCR values. The effect size of *X* was set to 0.5 standard deviations, a medium effect by conventional criteria (23). We repeated the simulations with smaller effect sizes, and there was no change in the qualitative conclusions.

## RESULTS

### Empirical data set

In the empirical data, the correlations between SB and qPCR LTL were very high at both time points: *r* = 0.90 (*P* < 0.001) for baseline and *r* = 0.85 (*P* < 0.001) for follow-up (Figures 1A and 1B). The correlation between SB and qPCR for ΔLTL was *r* = 0.48 (*P* = 0.03; Figure 1C). This was significantly lower than both the baseline (*z* = 2.77, *P* < 0.01) and follow-up (*z* = 2.25, *P* = 0.02) correlations.

The SB data showed a mean LTL shortening of 0.19 kilobases between baseline and follow-up, equivalent to 0.42 standard deviations of the baseline LTL variation. This would be considered significant shortening by conventional criteria, even in this small sample (*t* test against 0: *t* = −5.05, *P* < 0.001). By contrast, the qPCR data showed average LTL shortening of 0.12 standard deviations of the baseline LTL variation. This would be considered nonsignificant shortening by conventional criteria (*t* test against 0: *t* = −1.19, *P* = 0.25).

**Figure 2:**
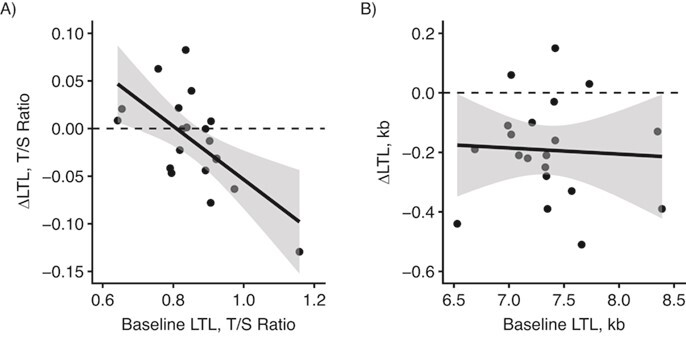
Relationship between change in leukocyte telomere length (ΔLTL) and leukocyte telomere length (LTL) at baseline in an empirical data set. A) Quantitative polymerase chain reaction (qPCR) data (*r* = −0.64, *P* = 0.003); B) Southern blotting (SB) data (*r* = −0.06, *P* = 0.82). Circles represent individual data points, black lines represent linear fits, and shading shows the 95% confidence intervals for the linear fits. In each case, the horizontal dashed line indicates the boundary between telomere shortening (below the line) and telomere lengthening (above the line). Blood samples were obtained from the Stony Brook University biorepository, Stony Brook, New York, 2012–2019. kb, kilobases; T/S ratio, ratio of telomere signal concentration (T) to that of the single-copy gene (S) (*36B4*).

In the qPCR data, ΔLTL depended negatively on baseline LTL (*r* = −0.64, *P* = 0.03, Figure 2A), while in the SB data, the correlation between ΔLTL and baseline LTL was weak and not significantly different from 0 (*r* = −0.06, *P* = 0.82; Figure 2B). The difference between these 2 correlations was significant (*z* = 2.04, *P* = 0.04). In the qPCR data, individuals with relatively short LTL at baseline tended to show LTL lengthening, while only those with relatively long LTL at baseline showed shortening. Of those individuals whose baseline LTL by qPCR was below the mean, 7 of 11 showed apparent lengthening, whereas of those whose baseline LTL by qPCR was above the mean, 8 of 9 showed apparent shortening. In the SB data, by contrast, 17 individuals showed shortening and only 3 showed apparent lengthening, with those 3 having neither particularly long nor particularly short baseline LTL.

### Simulated data sets

**Figure 3:**
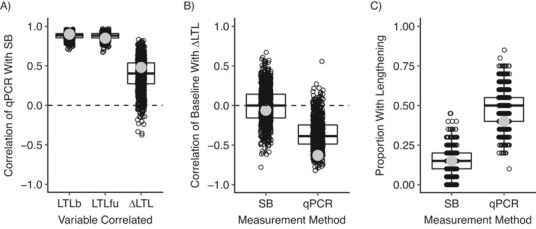
Properties of 1,000 simulated data sets (*n* = 20 for each one) drawn from distributions whose parameters matched those inferred from the empirical data. The box plots show median values and interquartile ranges. The small circles indicate individual simulated data sets. The larger gray circles indicate observed values from the empirical data. A) Correlations between quantitative polymerase chain reaction (qPCR) and Southern blotting (SB) for baseline leukocyte telomere length (LTL) (denoted LTLb), follow-up LTL (denoted LTLfu), and change in LTL (denoted ΔLTL); B) correlations between LTLb and ΔLTL for SB and qPCR; C) proportion of persons with apparent telomere lengthening for SB and qPCR. Blood samples were obtained from the Stony Brook University biorepository, Stony Brook, New York, 2012–2019.

We created 1,000 simulated data sets of *n* = 20 with the correlation between qPCR and SB at both baseline and follow-up set at 0.875 (the mean of the empirically observed baseline and follow-up values). We confirmed that in these data sets, the correlation between qPCR and SB for ΔLTL was always much lower than at either baseline or follow-up (median correlation, 0.42 (interquartile range (IQR), 0.26–0.55)). Similarly, the simulated data sets consistently showed marked signatures of measurement error in the qPCR data but not the SB data. There were consistently negative correlations between baseline LTL and ΔLTL for qPCR (median correlation, −0.38 (IQR, −0.50 to −0.25)) but not SB (median correlation, 0.01 (IQR, −0.15 to 0.18)). The percentage of persons with apparent telomere lengthening was higher for qPCR than for SB in 99.4% of simulated data sets (qPCR: median, 50% (IQR, 40%–55%); SB: median, 15% (IQR, 10%–25%)). The properties of the empirical data set fell well within the range of simulated data sets in all cases (Figure 3).

**Figure 4:**
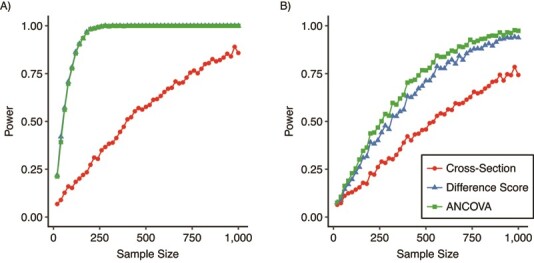
Statistical power to detect a true effect on telomere attrition (effect size *d* = 0.5) at *P* < 0.05, by analysis approach. Points represent observed power to detect a true effect of *d* = 0.5 from 1,000 simulated data sets. “Cross-section” ignores the baseline information and performs a cross-sectional analysis at the follow-up time point. “Difference score” treats change in leukocyte telomere length, the difference in leukocyte telomere length (LTL) between baseline and follow-up, as the outcome variable, whereas “ANCOVA” treats LTL at follow-up as the outcome variable and includes baseline LTL as a covariate. A) Simulated Southern blotting (SB) data (lines for difference score and ANCOVA are overlaid); B) simulated quantitative polymerase chain reaction data. ANCOVA, analysis of covariance. Blood samples were obtained from the Stony Brook University biorepository, Stony Brook, New York, 2012–2019.

We then took a range of sample sizes from 20 to 1,000 and simulated 1,000 data sets at each one. In these simulations, there was a true effect of a predictor variable on TL attrition. We assumed an effect size of *d* = 0.5. This would be conventionally considered a medium effect (23) and would lead to an 85–base-pair difference in LTL, on average, at follow-up. We plotted the statistical power to detect this true effect at *P* < 0.05 for each of the 3 analysis strategies, for the SB and qPCR data (Figure 4). For SB, there was a dramatic power gain from incorporating the baseline information (compare the power of the cross-section approach with the difference score and ANCOVA). For qPCR this power gain was much more modest. Note that the power levels of qPCR and SB for the cross-section approach were similar under these assumptions. However, the power for either of the longitudinal analyses was very much greater for SB than for qPCR. In addition, for SB, the powers of the difference score and ANCOVA approaches were almost identical, whereas for qPCR, there was a small but consistent power advantage for ANCOVA over the difference score.

## DISCUSSION

In this paper, we have presented results from the first data set (to our knowledge) in which LTL was measured at 2 time points in the same individuals by both SB and qPCR. The correlations between the 2 methods at both baseline and follow-up were high (*r* = 0.90 and *r* = 0.85); similar correlations in the past have been taken as validating that qPCR has sufficient precision for population studies (7–9). However, the correlation between SB and qPCR for ΔLTL, the change in LTL over the 6.6 years of the study, was only 0.48. Estimating change in LTL involves detecting a smaller range of true biological variation than estimating LTL in a cross-section, and it introduces twice as much measurement error. Thus, the correlation of a technique that has substantial measurement error with the gold standard is bound to be much lower when the target parameter is LTL change rather than LTL.

We also found that the qPCR data showed known signatures of measurement error much more strongly than the SB data. By qPCR, the apparent change in LTL depended negatively on LTL at baseline, and a substantial fraction of individuals showed apparent lengthening. These are predictable patterns due to regression to the mean in data sets containing measurement error (10, 16, 17). Again, simulations showed them to be consistently more marked in qPCR data sets where the cross-sectional correlations with SB are high but not perfect. Even with a small sample size (*n* = 20), we were able to detect significant LTL shortening over the 6.6 years of the study by SB. Indeed, the estimated rate (190 base pairs over 6.6 years implies 29 base pairs/year) accords well with previous estimates (10, 11). From the qPCR data, the researcher would have concluded that telomeres had not shortened, on average. Thus, although qPCR LTL estimates were highly correlated with SB estimates at both baseline and follow-up, relying on the qPCR data alone would have led to radically different conclusions about TL dynamics in this sample than using the SB data. Validating qPCR against SB cross-sectionally is therefore insufficient to infer that its precision is adequate for a longitudinal study of effects on TL dynamics.

The implications of our findings are as follows. First, since qPCR has not been validated as a measure of LTL for longitudinal use, only cross-sectionally, some skepticism may be in order about some published longitudinal findings obtained by qPCR. Most obviously, apparently null effects, like the apparent nonshortening with age in our empirical data set, may reflect false-negative findings due to low assay precision. However, there are also circumstances where the imprecision of qPCR carries substantial risks of false-positive findings as well. An effect of measurement error in longitudinal data, as we have demonstrated, is to make apparent LTL change strongly dependent on initial LTL. Any group of individuals whose LTL at baseline is relatively short will appear to show LTL lengthening, often of quite substantial magnitude. For example, in one study, Dershem et al. (24) concluded that gastric bypass surgery led to LTL lengthening in most cases. However, close examination shows that significant LTL lengthening was restricted to those individuals whose baseline LTL was the shortest (25). In another study, Meier et al. (26) concluded that persons with the greatest exposure to chronic stress showed the least LTL shortening over 10 years. Again, these individuals were also the ones with the shortest LTL at baseline. We emphasize that our empirical qPCR assays were performed at a laboratory whose staff were experienced in the field, and the cross-sectional correlations with SB were at the high end of what has been previously published. There are grounds for believing that many published qPCR data sets have much lower precision than was observed here (11, 16). It is likely, therefore, that the issues we document are present and perhaps even more severe in the published literature.

Second, researchers using qPCR have a number of options for reducing the effective measurement error: increasing the number of technical replicates; making specific corrections for plate and well position; ensuring consistency of sample storage and DNA extraction and integrity; and controlling for variable amplification efficiency. These options have been discussed in detail elsewhere (7, 16, 27–30). Since the consequences of a small amount of imprecision are more dramatic for TL change than they are for TL itself, the point of diminishing returns in the application of these measures may actually be much higher than researchers appreciate. The fact that qPCR data correlate with a gold standard at *r* = 0.90 for a cross-section may appear “good enough,” and, indeed, that may be so in cross-sectional analyses. However, as we have shown, if the goal of the study is to detect subtle changes in TL over time, it may not be. In the simulations underlying Figure 4, raising the cross-sectional correlation to *r* = 0.95 has a quite dramatic effect on the power of qPCR for the difference score and ANCOVA.

Third, our statistical power simulations have implications for optimal choices of telomere measurement method and data analysis strategy. The cross-section analysis strategy used in our simulations makes no use of the baseline information and thus captures the power consequences of performing a purely cross-sectional study. The other 2 strategies represent different ways of incorporating longitudinal information. Our simulations show that, for SB data, there is a dramatic power advantage for performing longitudinal analysis. As Aviv et al. (4) have argued elsewhere, for SB, within-individual, longitudinal comparison will provide much increased sensitivity for detecting factors that affect TL dynamics, even if the expense is considerable and the resulting sample size is smaller. For qPCR, the power gain from longitudinal comparison, though still present, is much more modest, as our simulations show. The large gain from performing within-individual comparisons is partially offset by the loss of introducing a second set of measurement errors. We note, of course, that increasing statistical power is not the only motive for choosing a longitudinal analysis. There are others, such as eliminating reverse causality (2).

Simply put, the statistical power of qPCR may be nearly as high as that of SB for a cross-sectional study. So, if using qPCR makes a larger sample size possible, it could represent a beneficial decision. From the simulated data in Figure 3, the power of a cross-sectional qPCR study with 500 individuals is better than the power of an equivalent SB study with 250 individuals. However, the researcher planning a longitudinal analysis might usefully consider using SB or another precise method, even if that will entail a much smaller achieved sample size. In our simulations, for either of the longitudinal analyses, the power of a qPCR study with 1,000 individuals is still worse than that of an equivalent SB study with 250. Thus, optimal decisions about TL measurement method and sample size are linked to those about study design and objectives.

We also noted some differences between the statistical powers of the 2 longitudinal data analysis strategies, difference score and ANCOVA. For SB, the power of these 2 strategies is almost identical. For this reason, researchers should choose the difference score, since ANCOVA introduces collider bias if baseline LTL differs by the predictor variable, whereas the difference score is not vulnerable to such bias (14). The similar power of the 2 approaches by SB accords with previous simulations, which showed that the power of the difference score and the power of ANCOVA converge when the correlation between baseline and follow-up measurements is high (13). For qPCR, ANCOVA provides a small but consistent power advantage over the difference score. Thus, ANCOVA should be used as long as the association between the predictor variable and baseline LTL is close to 0 (i.e., the judgment should be made on the basis not of statistical significance but the size of the parameter estimate). If there is any nonzero association, the difference score method should be selected in order to avoid collider bias (and baseline LTL should not be included as an additional covariate, as is sometimes seen) (14). Note that researchers sometimes fit mixed models with LTL as the outcome variable, testing whether there is an interaction between a time point and the predictor variable. This is equivalent to using the difference score from the standpoint of power and collider bias (14).

For our statistical power simulations, we treated SB as if it directly reflected the true TL. This makes sense in as much as we were using SB here as a gold standard against which qPCR could be validated. In reality, however, SB too involves some measurement error, and the true TLs are not known. Thus, our simulation results should be taken as reflecting the *relative* statistical powers of SB and qPCR for different analysis strategies (or, indeed, the statistical power of qPCR relative to any gold standard the researcher might use). The absolute values of the SB power given may be overestimates. Moreover, associations in telomere epidemiology tend to be much weaker than the association illustrated in Figure 4 (1). The simulation code provided in conjunction with this paper (22) allows users to rerun the simulations for different effect sizes.

In conclusion, demonstrating that a measurement method captures much of the same variation in TL as the gold standard method in a cross-sectional study does not entail that it will capture the same variation in TL change in a longitudinal one. We have shown empirically that in a data set where the cross-sectional correlations between qPCR and SB are high at both baseline and follow-up, agreement between the 2 methods for TL change over time is fairly poor and the qPCR data show stronger signatures of measurement error than the SB data. As we have shown, this has implications for investigators choosing how to allocate their research resources to a suitable combination of study design, measurement method, sample size, and data analysis strategy.
